# Toxic epidermal necrolysis caused by viral hepatitis A: a case report and literature review

**DOI:** 10.3389/fmed.2024.1395236

**Published:** 2024-06-05

**Authors:** Yun Ye, Qian Zhang, You-Wen Tan

**Affiliations:** Department of Hepatology, The Third Hospital of Zhenjiang Affiliated Jiangsu University, Zhenjiang, Jiangsu, China

**Keywords:** toxic epidermal necrolysis, hepatitis A virus, treatments, skin, immune-mediated

## Abstract

Toxic epidermal necrolysis (TEN) is a rare but serious immune-mediated life-threatening skin and mucous membrane reaction that is mainly caused by drugs, infections, vaccines, and malignant tumors. A 74-year-old woman presented with a moderate fever of unknown cause, which was relieved after 2 days, but with weakness and decreased appetite. Red maculopapules appeared successively on the neck, trunk, and limbs, expanding gradually, forming herpes and fusion, containing a yellow turbidous liquid and rupturing to reveal a bright red erosive surface spreading around the eyes and mouth. The affected body surface area was >90%. The severity of illness score for toxic epidermal necrolysis was 2 points. The drug eruption area and severity index score was 77. She was diagnosed with TEN caused by hepatitis A virus and treated with 160 mg/day methylprednisolone, 300 mg/day cyclosporine, and 20 g/day gammaglobulin. Her skin showed improvements after 3 days of treatment and returned to nearly normal after 1 month, and liver function was completely normal after 2 months.

## Introduction

Stevens–Johnson syndrome (SJS) is an uncommon but severe immune-mediated skin disorder. Toxic epidermal necrolysis (TEN) is an exacerbated manifestation of SJS, affecting more than 30% of the total body surface area (1). Its main causes include drug use and infections. Drugs that cause TEN include non-steroidal anti-inflammatory, aromatic anti-epileptic, anti-gout, antibacterial drugs, biological agents, and proprietary Chinese medicines. The related pathogens are *Mycoplasma* spp. and herpes simplex viruses. Human herpes virus (HHV)-6 (1), Epstein–Barr virus (EBV) (2), *Mycoplasma pneumoniae* (3–5), and novel coronavirus infection (6, 7) are associated with the occurrence and progression of SJS/TEN. *M. pneumoniae* and novel coronaviruses can directly cause SJS/TEN. Although TEN caused by viral hepatitis is rare, we report a new case of TEN caused by the hepatitis A virus with severe liver function impairment.

## Case presentation

A 74-year-old woman developed fever with a maximum temperature of 38.4°C. She did not presented the symptoms cough, runny nose, or sore throat, and was not treated with any drugs. Two days later, her body temperature returned to normal; however, she developed fatigue and nausea, and a maculopapular rash appeared on her face, neck, chest, and back without pain. On Day 3, the fatigue symptoms worsened, appetite remained poor, and erythema of the trunk and limbs continued to expand progressively. On Day 5, the rash increased gradually and became herpetic, merging gradually into the bulla with partial epidermal exfoliation and skin tearing. After dermatological consultation, the patient was admitted to the hospital for the treatment of TEN.

At the physical examination on admission, body temperature, pulse, blood pressure, and respiration was 37.4°C, 82 beats/min, 137/82 mmHg, 20 breaths/min, respectively; the breathing sound was clear in both lungs without dry and wet rales in the lower lungs and cardiac rhythm was regular with no pathological murmurs. The mental condition was slightly poor; the patient could answer questions correctly but was uncooperative with the physical examination. Dermatological examination revealed multiple erythematous lesions, maculopapules, bullosa of the trunk and limbs, erythema fused into pieces, positive Nishler’s sign, bullovesicular wall relaxation, and yellow turbidous fluid. The vulva, perianal fold area, and compressed parts of the large area of collapse revealed a bright red erosive surface with evident seepage. The skin around the eye and external ear canal were broken, conjunctiva was congested, and eye was slightly photophobic. The lips were covered with dark red crusting that was bleeding; however, the oral mucosa remained unaffected. The affected body surface area was >90%, severity-of-illness score for toxic epidermal necrolysis (SCORTEN) (8) was 2 (age > 40 years, 1 point; epidermal exudation area was >10% of the total surface area, 1 point), drug eruption area and severity index (DASI) (9) score was 77, and Zubrod–ECOG–WHO physical status score was 3.

Blood tests 6 days after the initial fever showed a white blood cell count of 12.9 × 10^9^/L [reference value: (3.5–9.5) × 10^9^/L], lymphocyte ratio of 0.42 (0.200–0.500), lymphocyte count of 4.9 × 10^9^/L [(1.1–3.2) × 109/L], neutrophil ratio 0.694 (0.400–0.750), neutral granulocyte count of 7.9 × 109/L[(1.8–6.3) × 10^9^/L], and eosinophil count of 0.23 × 10^9^/L [(0.02–0.52) × 10^9^/L]. The blood biochemistry was as follows: creatinine 77 μmol/L (59–104 μmol/L), urea 4.3 mmol/L (2.8–7.1 mmol/L), uric acid 344 μmol/L (89–420 μmol/L), and albumin 34.6 g/L (35.0–50.0 g/L). The patient was prescribed 160 mg/day methylprednisolone, 300 mg/day cyclosporine, 20 g/day gamma globulin, and external aureomycin eye cream twice daily for both eyes and face to prevent adhesion.

**Figure 1:**
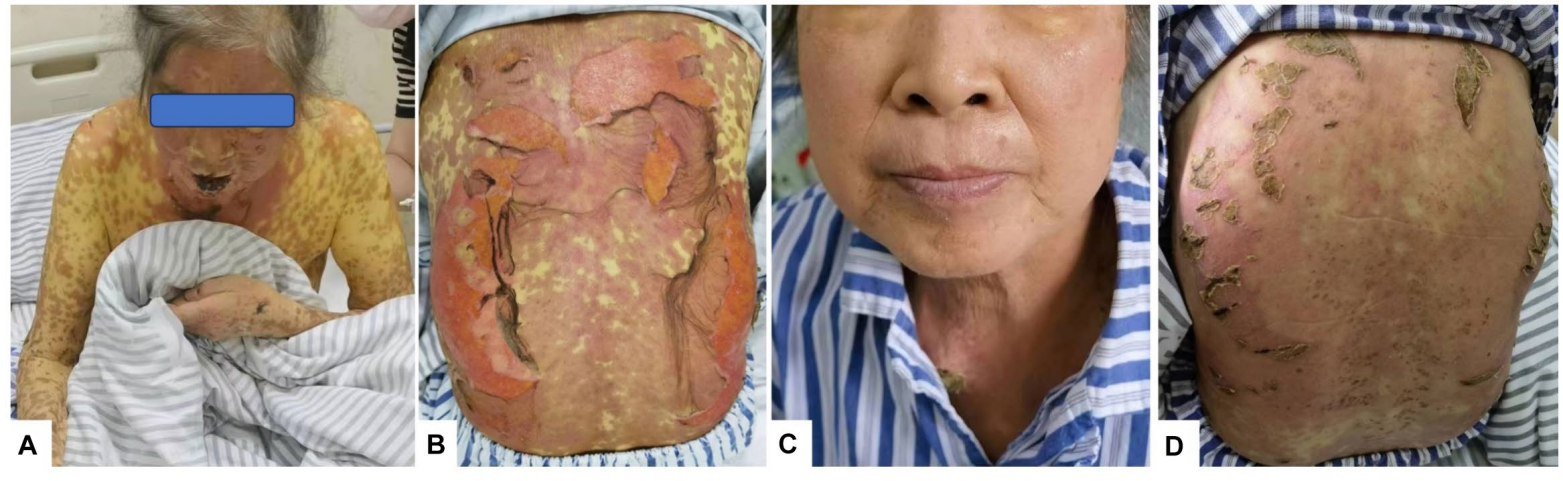
Rash changes. **(A)** On Day 10 of the disease course, the skin on the face and arms began to peel, forming scabs. **(B)** On Day 12 of the disease course, lesions on the back broke, exfoliated, and oozed. **(C)** On Day 26 of the disease course, facial skin appeared normal. **(D)** By Day 26, the skin on the back had healed and formed crusts.

One week later, the skin lesions on the exposed parts of the patient’s face, torso, limbs, and other areas continued to improve, the skin in the areas of compression and folding was dry and healed gradually, but the pigmentation remained (Figure 1A); the DASI score was 42.8, no new maculopapules appeared on the limbs, and herpes absorption was observed. However, the back still had a large area of ulceration and exudation with pain (Figure 1B), fatigue improved, and appetite remained poor. Therefore, cyclosporine and immunoglobulin were discontinued and the methylprednisolone dose was reduced to 60 mg/day.

**Figure 2:**
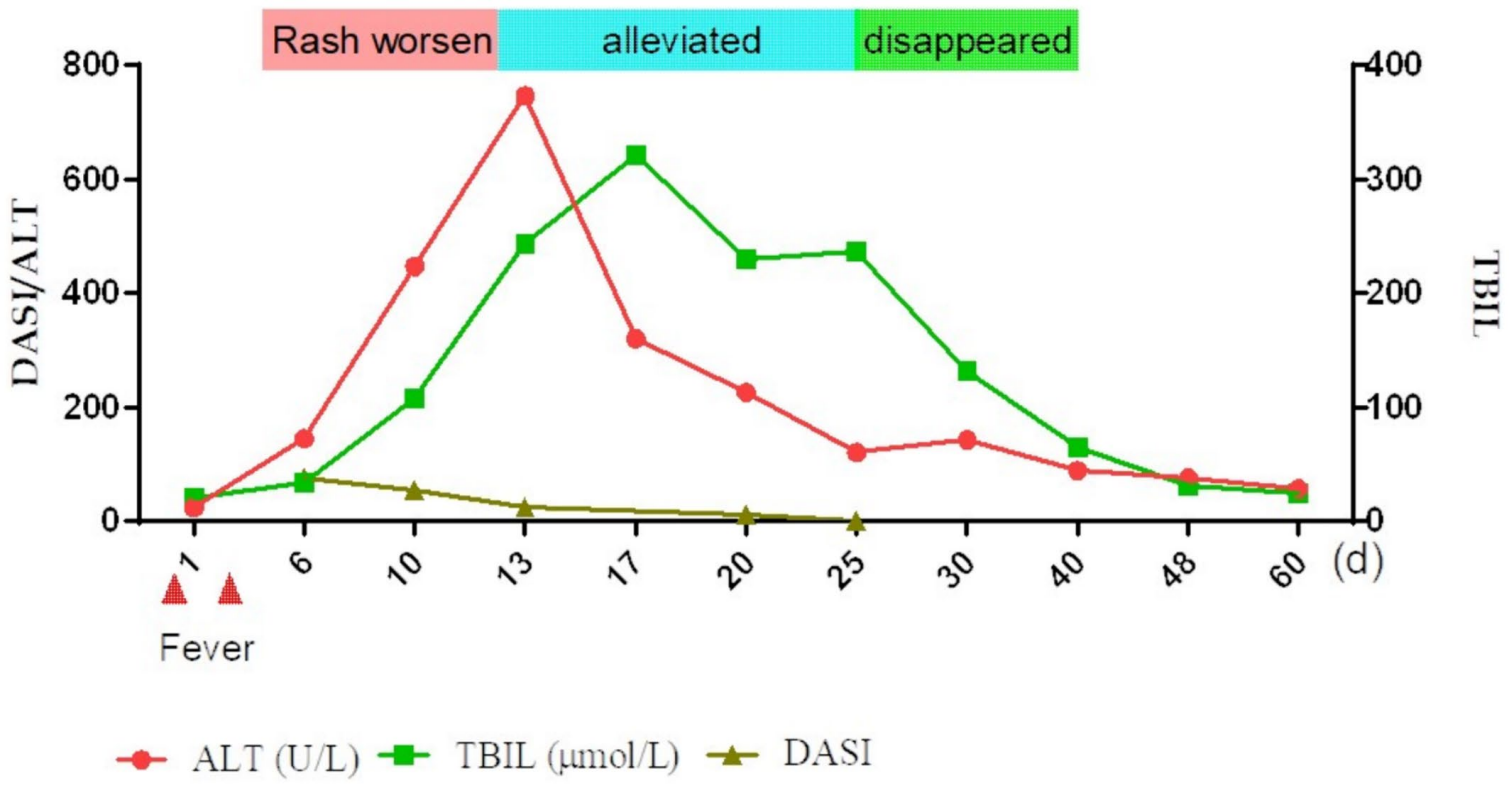
Overall brief disease flowchart. TBIL, total bilirubin; ALT, alanine aminotransferase; DASI, severity index.

Nine days after the initial fever, liver function was significantly abnormal: total bilirubin (TBIL) 34.4μmo1/L, direct bilirubin (DBIL) 24.2 μmol/L, aspartate aminotransferase (AST) 146 U/L, alanine aminotransferase (ALT) 145 U/L, alkaline phosphatase (ALP) 209 U/L, glutamyl aminotransferase (GGT) 414 U/L, and prothrombin time 13 s. Prothrombin activity was 98% and the international normalized ratio (INR) was 1.12. The anti-HAV-IgM test results were strongly positive. An infectious disease physician diagnosed acute hepatitis A (AHA) and transferred the patient to the infectious disease department for treatment with adenosine, methionine, and ursodeoxycholic acid. The patient’s back was still ruptured and exudated, but other parts had improved gradually; however, the liver function indexes continued to deteriorate even a week later (TBIL 243.2 μmo1/L, ALT 746 U/L, ALP 674 U/L, GGT 1387 U/L). The skin had improved significantly on October 5 (Figures 1C,D). Therefore, the methylprednisolone treatment was discontinued. An overall disease progression flowchart is shown in Figure 2. Tests for Epstein–Barr virus (EBV), adenovirus, influenza A and B viruses, and SARS-CoV-2 were negative. Liver function indicators improved gradually and were completely normal after 2 months, with no alcohol consumption, history of other drug use or toxic exposure within 3 months before hospitalization, and history of allergy, B-E viral hepatitis markers, or anti-mitochondrial antibodies. Tests for antinuclear antibodies, anti-smooth muscle antibodies, anti-liver and anti-kidney microsomal antibodies, and other autoantibodies yielded negative results. The patient was diagnosed with TEN caused by hepatitis A virus (HAV).

## Discussion

TEN is a serious, life-threatening cutaneous mucosal reaction that can occur at any age. The main causes are drug use and infections. The typical clinical manifestations include extensive skin/mucosal erythema, blisters, and severe epidermal necrolysis. This disease progresses rapidly. Multiple organs may be involved, with high mortality rate (14.8–48%) (10). TEN is a severe rash that must be differentiated from other types of rashes (Table 1). TEN pathogenesis involves T cell-mediated type IV hypersensitivity triggered by stimulants, such as drugs, with the cytokines associated with its pathogenesis serving as suitable biomarkers. Specific biomarkers, including galectin-7 (11) and receptor interaction protein 3 (12), and non-specific biomarkers, such as granolysin (13), chemokine ligand 27 (14), and soluble apoptosis-related factor ligand, contribute to the early diagnosis of TEN (15). However, no biomarkers with high specificity for pathogenic factors have been identified so far. In this patient, the dermatologist initially considered the cause to be drug-induced; however, a careful inquiry into the medical history showed no history of toxicant or drug exposure, and no suspected causative agents were found in the environment. Infection is considered another important cause of TEN; human herpesvirus (HHV)-6, EBV, *M. pneumoniae*, and novel coronavirus infections are associated with the development of TEN (16). The patient initially presented with a brief fever that resolved after 2 days. We considered this infection to have caused TEN. We observed that common respiratory viruses and bacteria were not the causative agents, and the positivity for anti-HAV-IgM was unexpectedly strong (three tests within 1 month). Positive anti-HAV-IgM is insufficient evidence to diagnose AHA; however, the patient had symptoms, such as fever, fatigue, and decreased appetite, accompanied by significant abnormalities in liver function; after ruling out other common causes of liver damage, the final diagnosis was AHA.

**Table 1:** Key differences among the types of severe rash.

	TEN	EM	SSSS	AGEP	DRESS
Favorite group	Adult	Children and young women	Infant, occasionally in adults	Adult	Adult
Common causes	Drugs, infections	Infections, drugs, and diseases	*Staphylococcus aureus*	Antibacterial drugs (β-lactam, macrolides), nonsteroidal anti-inflammatory Drugs.	Medication, human herpesvirus 6
common symptom	Fatigue, chills, myalgia, and fever	Headache, fever, joint and muscle aches, tonsillitis, and respiratory infections;	Impetigo, scalded bulla, exfoliation of large epidermis	The appearance of non-follicular sterile pustules is preceded by erythema, often accompanied by fever and other symptoms.	The clinical manifestations are diverse, with a long incubation period (2–8 weeks), often manifested as fever, lymph node enlargement, increased eosinophils, and multiple organ damage
Characteristics of rash	Painful local erythema that spreads quickly with a loose bulla or peeling of the epidermis on the erythema. Extensive peeling, extensive erosion, including all mucous membranes (eyes, mouth, external genitals) if lightly touched or pulled	The rashes are polymorphic, with erythema, papules, wind masses, blisters, bullosa and purpura, etc. The rashes of this disease are polymorphic, with typical target-shaped lesions, which tend to occur at the extremities, symmetrical distribution, mucosal lesions, and systemic symptoms, such as fever in severe cases	The disease appears suddenly, with erythema occurring around the mouth or eyelids at first, and then rapidly spreading to the trunk and proximal limbs, or even generalized throughout the body, with obvious pain at the lesions. Laxative bullae occur based on erythema, and exudation and scabs occur around the mouth and eyelids within 1 to 2 days, with large areas of scabs falling off, leaving radiating clefts around the mouth.	The rash typically initiates on the facial area and in skin folds, such as the neck, armpits, groin, etc., and rapidly disseminates to encompass a large portion of the skin within a short timeframe. The rash presents as superficial sterile pustules, small and densely distributed, accompanied by a certain burning or itching sensation.	Facial edema, rapidly affecting erythema and papules throughout the body, can also manifest as purpura, exfoliative dermatitis, etc.
Treatment	Cyclosporin A, gamma globulin, glucocorticoid	Etiological treatment	Adequate and effective antibiotics should be used in the early stages	Discontinue suspicious drugs and use glucocorticoid	Discontinue suspicious drugs and use glucocorticoid

TEN, toxic epidermal necrolysis; EM, erythema multiforme; SSSS, staphylococcal scalded skin syndrome; AGEP, acute generalized exanthematous pustulosis; DRESS, drug rash with eosinophilia and systemic symptoms.

Hepatitis A, caused by HAV, is a self-limiting disease that is mainly transmitted via the fecal–oral route (17). China established a national self-funded vaccination program from 1992 to 2002 (18), including live attenuated and inactivated hepatitis A vaccines. The program effectively reduced the number of people susceptible to hepatitis A, and the incidence of hepatitis A decreased from 55.7 per 100,000 people in 1991 to less than 2 per 100,000 people in 2017. The hepatitis A epidemic in China has changed gradually from a high to a medium–low level (19). Hepatitis A infections are sporadic. In non-endemic areas, the diagnosis of hepatitis A is no longer dependent on the detection of HAV RNA, but on positive anti-HAV-IgM, clinical manifestations, and biochemical indicators of liver injury, such as abnormal ALT and TBIL levels.

Skin manifestations of AHA are rare. A transient rash occurs before or simultaneously with an acute illness (20). The rashes observed are usually macular erythema, papular rash, and, less commonly, urticaria, purpura, or petechia (21). A few other cases of unusual rashes, including Gianotti–Crosti syndrome, Henoch–Schönlein purpura (22), and cutaneous vasculitis, have been reported (23) (Table 2). HAV-induced TEN caused by HAV is rare. In 1989, Werblowsky–Constantini (39) first reported the case of a 35-year-old man with fever for 3 weeks, 50% skin lesions, hyperbilirubinemia, and high ALP levels, similar to our patient’s liver function. Zang et al. (40) also reported a case of TEN related to HAV infection in a 38-year-old male with cirrhosis and preexisting liver failure. The patient developed skin lesions 15 days after the TBIL reached its peak, accompanied by fever with a maximum body temperature of 39.0°C, and sustained remission was achieved with intravenous corticosteroids.

**Table 2:** Dermatological manifestations of hepatitis A infection.

Dermatologic manifestations
Common manifestations
Urticaria (20, 24)
Scarlatiniform eruption (20, 24)
Serum sickness-like illness rash (21, 24)
Evanescent skin rash (21, 24)
Maculopapular prolonged rash (24)
Uncommon manifestations
Porphyria cutanea tarda (25)
Lichen planus (26)
Guillain-Barré syndrome (27, 28)
Immune thrombocytopenic purpura (29)
Systemic lupus erythematosus (30–32)
Rare manifestations
Cryoglobulinemia (33, 34)
Vasculitis (35)
Primary hepatic lymphoma (36, 37)
Henoch-Schönlein purpura (28, 38)
Toxic epidermal necrolysis (39, 40)

It is a self-limiting disease that rarely causes liver failure. In our case, rapid deterioration of liver function occurred, especially with a progressive increase in total bilirubin and ALT levels, but no symptoms, such as high fatigue, ascites, or confusion, occurred. The prothrombin time and INR indices were normal, and acute liver failure could not be diagnosed. Multiple organ injuries often involve the liver and kidneys. Moreover, TEN can damage multiple organs, including the liver and kidneys. SJS/TEN can also be used to assess severity according to SCORTEN (8) and predict mortality. The score included the following seven factors, with one point for each risk factor: age > 40 years, complications of malignant tumor, exfoliation area > 10% of the total surface area, heart rate > 120 beats/min, serum urea nitrogen >10 mmol/L, venous blood glucose >14 mmol/L, and blood bicarbonate level < 20 mmol/L. The total score was 0–7 points, and the corresponding predicted mortality rates were 3.2% (0–1 point), 12.1% (2 points), 35.8% (3 points), 58.3% (4 points), and 90.0% (>5 points). The patient’s mean score was 2 points. Currently, no specific drug has been established for the treatment of SJS/TEN. However, supportive therapy can confer tangible benefits. Using systemic corticosteroids remains contentious, as initial observational studies have indicated markedly elevated rates of infection, including *Candida* sepsis and overall complications, leading to higher mortality (8) in patients receiving corticosteroid treatment. In contrast, recent investigations have proposed a survival advantage for patients treated with corticosteroids compared with those receiving supportive measures (41) alone.

A recent systematic review and meta-analysis (42) including 96 studies (3,248 patients) reported that the applied therapies included supportive or systemic immunomodulatory therapies, such as glucocorticoids, intravenous immunoglobulin, cyclosporine, plasma exchange, thalidomide, cyclophosphamide, hemoperfusion, tumor necrosis factor inhibitors, and granulocyte colony-stimulating factor. Glucocorticoids were associated with survival benefits in all three analyses but were statistically significant in only one analysis. Despite the small number of patients, cyclosporine was associated with promising significant results only in the feasibility analysis of the unstratified models (OR, 0.1; 95%CI, 0.0–0.4). No beneficial effects were observed with other therapies, including intravenous immunoglobulin.

This study had certain limitations. First, the patient was not tested for *M. pneumoniae* in the present study. Although patients do not develop respiratory symptoms, TEN is common. However, this possibility cannot be excluded. Second, no skin biopsy was performed, which is of great diagnostic significance. Third, HAV RNA was not detected in the present study. Finally, although the patient did not take any drugs, the possibility of drug-induced TEN could not be ruled out because trace amounts of drugs, especially antibiotics, may be present in certain foods.

In conclusion, we present a case of TEN caused by hepatitis A virus infection and severe liver function injury. Although drugs remain the primary pathogenic factors for TEN, in cases where physicians are unable to obtain a clear history of drug use and exposure to sensitizing substances, biological factors, including the uncommon pathogenic viruses and bacteria associated with TEN, must be considered. Furthermore, when there is evidence of organ damage, rare pathogenic factors, such as the liver injury observed in this case, must be investigated; this led us to believe that the hepatitis A virus was the causative agent. This case report also serves as a reminder to infectious disease specialists that hepatitis A virus infection may lead to rare severe skin reactions that can be effectively managed with early corticosteroid treatment, resulting in satisfactory recovery.

## Data availability statement

The original contributions presented in the study are included in the article/supplementary material, further inquiries can be directed to the corresponding author.

## Ethics statement

The requirement of ethical approval was waived by The Third Hospital of Zhenjiang Affiliated Jiangsu University for the studies involving humans because The Third Hospital of Zhenjiang Affiliated Jiangsu University. The studies were conducted in accordance with the local legislation and institutional requirements. The participants provided their written informed consent to participate in this study. Written informed consent was obtained from the individual(s) for the publication of any potentially identifiable images or data included in this article. Written informed consent was obtained from the participant/patient(s) for the publication of this case report.

## Author contributions

YY: Investigation, Writing – original draft. QZ: Data curation, Investigation, Writing – review & editing. Y-WT: Project administration, Writing – original draft, Writing – review & editing.
